# Evaluation of thermal camera measurement stability and factors associated with infrared eye temperature in dogs

**DOI:** 10.3389/fvets.2026.1848874

**Published:** 2026-05-26

**Authors:** Sahar Rostami, Kimberly A. Woodruff, Abigail McBride, Jingyi Shi, David R. Smith

**Affiliations:** 1 Department of Pathobiology and Population Medicine, College of Veterinary Medicine, Mississippi State University, Starkville, MS, United States; 2 Department of Mathematics and Statistics, College of Arts and Sciences, Mississippi State University, Starkville, MS, United States

**Keywords:** canine, eye temperature, infrared thermography, rectal temperature, shelter dogs

## Abstract

Non-invasive thermal monitoring, such as infrared thermography (IRT), offers a potential alternative to traditional thermometry in veterinary settings. This study aimed to characterize the relationship between maximum eye temperature (ET) and rectal temperature (RT) in dogs. Our field study included 97 observations from 57 shelter dogs whose ET was measured using IRT on 4 separate days. For each observation, 3 consecutive ET measurements were obtained at 10-min intervals, followed by a RT measurement. Device temporal stability was evaluated measurement drift using an insulated box as a controlled environment; the temperature of a fixed point on the inner wall was recorded at 5-min intervals for 1 h using the same thermal camera. Data from both studies were analyzed using repeated-measures linear mixed-effects regression models. In field study, RT, age group, and repetition number were significantly associated with ET. For every 1 °C increase in RT, ET increased by an estimated 0.6 °C (95% CI, 0.3 to 0.9). On average, compared with puppies, adults had lower ET (*β* = −0.4 °C; 95% CI, −0. 6 to −0.1), and ET decreased with repeated measurements. Device Stability assessment demonstrated a consistent drop in temperature readings, suggesting information bias related to the camera. Eye temperature measured by IRT was positively associated with RT, and age-related differences were observed. Repeated measurements showed a progressive decline in ET, consistent with device-related measurement drift rather than physiologic change. These findings underscore the importance of accounting for equipment performance characteristics when considering application of IRT. Eye thermography may serve as a noninvasive adjunct for assessing body temperature in dogs; however, device-related behavior should be validated and considered when interpreting readings.

## 1 Introduction

Body temperature is routinely used as a physiologic marker of health and can change with inflammation, systemic illness, environmental disturbances or stress (1–3). Timely identification of abnormal body temperature in dogs, might support early clinical evaluation and, in group-housed setting, might assist with infectious-disease risk managements (4–6). Traditional body temperature measurement methods, such as rectal thermometry, present several drawbacks. Rectal temperature (RT) measurement can be time-intensive, requires restraint, and may increase stress in some dogs. Measured RT can also be affected by factors such as rectal contents and may not reflect rapid changes in body temperature. Because the method involves contact, infection-control procedures are needed to minimize the risk of indirect transmission via equipment between animals (7–9). Shared thermometers might contribute to indirect pathogen transmission when disinfection protocols are inconsistent (10). This limitation underscores the need for non-invasive, reliable tools for routinely monitoring body temperatures of animals.

Infrared thermography (IRT) has increasingly been adopted in veterinary diagnostics because it enables rapid, contact-free assessment of superficial thermal patterns associated with perfusion and inflammation (11). This non-contact method is valuable because sudden changes in skin or eye heat are often caused by increased blood flow due to underlying inflammation. These thermal changes can serve as an early indication of sickness, even before the animal displays visible clinical signs (12–14). Recent studies have demonstrated its utility in detecting physiological changes, inflammation, and heat stress in various species, including dogs (15, 16). IRT measures surface temperatures from specific anatomical regions, such as the eye, ear, and gum, which have shown varying degrees of correlation with body temperature (17). Zanghi et al. demonstrated that ear and eye temperatures (ET), measured using IRT, were significantly correlated with RT during physical activity, with ear temperature proving more reliable for detecting hyperthermia (18). Similarly, a study involving Kangal dogs reported a significant positive correlation between rectal, ear, and eye temperatures (19). When agreement analysis was utilized in a separate study, researchers found that while ET fluctuated alongside RT, ET readings consistently underestimated RT meanwhile, gingival temperature had the lowest mean difference with RT. (20) However, measuring gingival temperature in dogs still requires direct contact with the animal, which can be a limitation in terms of stress, cooperation, and potential discomfort for the dog. Beyond temperature screening, IRT has also been used as a non-invasive indicator of stress and welfare-related physiologic responses in animals (8, 21). The reliability of thermographic readings depends on the anatomical site selected, local tissue perfusion and vascularization, hair coat characteristics, and environmental conditions such as ambient temperature, humidity, and airflow (22–25). The objective of this study was to evaluate ocular thermography under practical field conditions. Recognizing that the eye and the rectum are distinctly different anatomical sites, our goal was not to establish an exact prediction formula or test for perfect diagnostic agreement. Instead, we aimed to determine whether surface ET naturally fluctuates in relation to changes in RT, helping us assess the diagnostic utility of IRT as a fast, non-invasive temperature evaluation tool. We also sought to identify the biological traits and device-specific factors that influence these non-contact readings. By taking this real-world approach, we aim to provide a realistic foundation for understanding both the limitations and the true value of thermal cameras as a fast, restraint-free triage tool.

## 2 Materials and methods

### 2.1 Field study: ocular thermography in shelter dogs

The first part of this study was conducted in an animal shelter. Data collection was conducted over 4 days, with measurements taken at approximately the same time each afternoon to minimize variability due to circadian rhythms. For each dog, sex, age category (puppy vs. adult), and body size category (small, medium, or large) were recorded. Dogs were eligible if they had normal clinical signs on visual inspection at enrollment (such as, no obvious signs of systemic illness and no excessive tearing and nasal secretions). Animal-use oversight was provided by the Mississippi State University Animal Care and Use Committee, and authorization to enroll shelter-owned dogs was obtained from shelter management.

#### 2.1.1 Thermal camera measurements

All thermal camera measurements were performed indoors with the dogs remaining unrestrained in their kennels. Ambient temperature and humidity were recorded using an INKPET thermometer (accuracy: ±1.0 °C) and hygrometer (accuracy: ±5%RH) and entered into the camera settings. Thermal images were obtained with a thermal camera (Fotric 326 M; thermal resolution, 384 × 288 pixels; thermal sensitivity, 40 mK [0.04 °C]). The emissivity was set to 0.98, as recommended by the camera manual and we ensured 15 min warm-up period before recording temperatures to achieve optimal performance as recommended by the camera operating manual.

**Figure 1:**
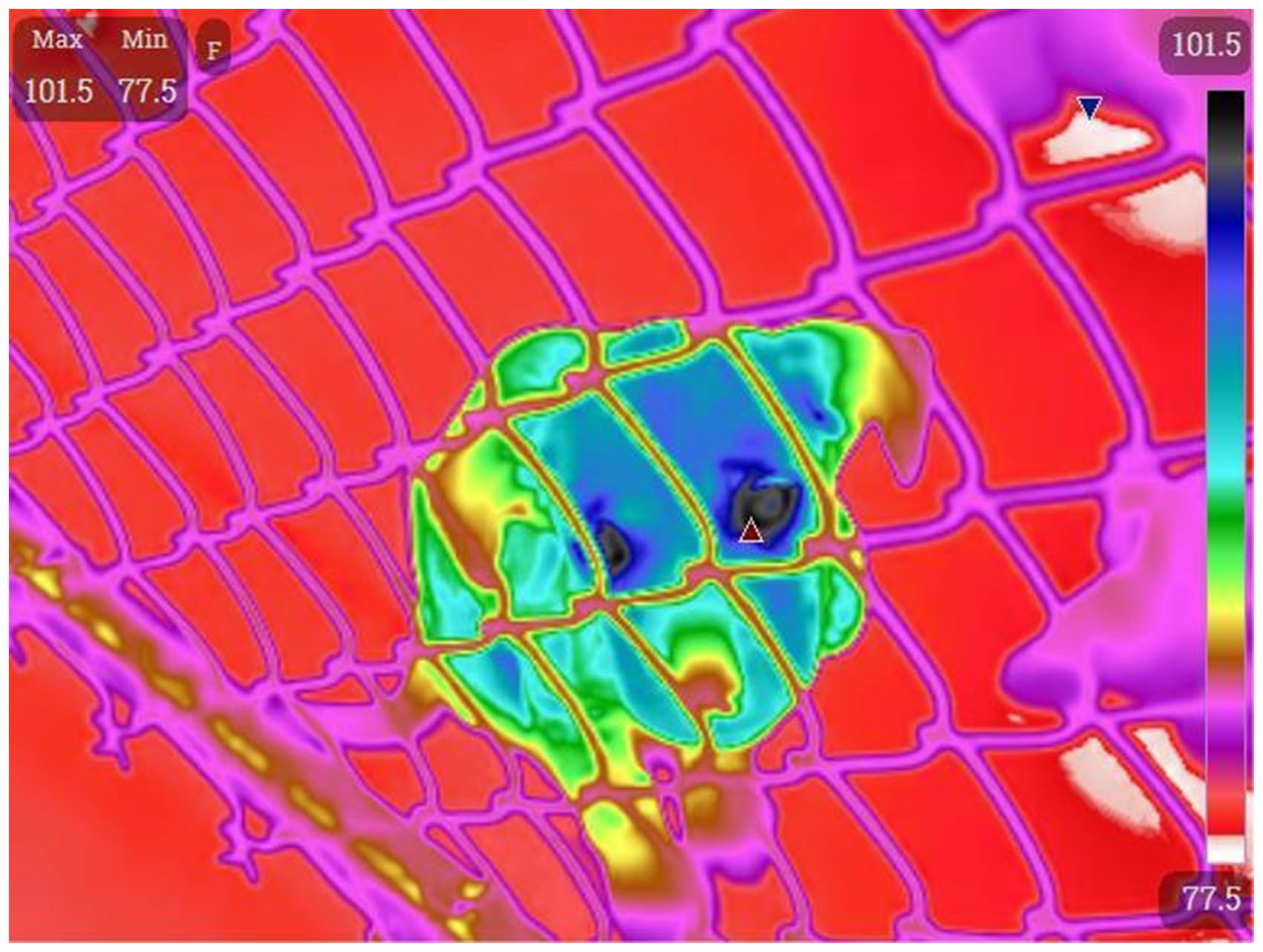
Thermal image obtained from a mixed-breed female puppy housed in a shelter kennel. The maximum eye temperature (ET) was recorded by evaluating both eyes and selecting the highest value obtained (either the left or right eye).

Eye temperature readings were taken three times per dog, with about 10 min interval between each reading (time = 1, 2, and 3). The camera was positioned approximately 60 cm from the dog’s face to capture thermal images. Images were analyzed using AnalyzIR Mercury software using this software, we drew a region of interest around the visible areas of both eyes. The software automatically identified the single highest pixel temperature within those regions. Temperature readings were taken in Fahrenheit and subsequently converted to Celsius for reporting and for each time point, the maximum temperature from either eye was used for analysis (Figure 1).

#### 2.1.2 Rectal temperature measurements

Rectal temperature measurements at the same depth were conducted in the same environment where ET readings were taken. After completing the three ET readings for all enrolled dogs each day, one RT reading was measured using a digital rectal thermometer. Animals were calmly held by the authors during data collection to ensure safety and comfort. All measurements were conducted in a rapid, calm manner to minimize the duration of handling and prevent stress-induced fluctuations in body temperature. Lubricant was applied to the thermometer before inserting into the rectum to minimize discomfort and ensure accurate readings.

After the shelter data collection, we noted a consistent decline across the 3 serial ET measurements within dogs; therefore, Experiment 2 was performed to evaluate thermal camera output over time under controlled conditions.

### 2.2 Assessment of device stability

This part of the study was designed as a follow-up assessment to determine if a decreasing trend observed during the sequential temperature captures in field study was related to instrument-related drift. We constructed a controlled test system using two insulated shipping containers (each approximately 39 cm 32 cm, 30 cm) taped together and interior wall, lined with paper. Emissivity was set to 0.93 based on camera manual. Emissivity adjustments were implemented to minimize measurement error and ensure that recorded temperatures accurately reflected the surface radiance of the specific target being imaged. This system was placed in a stable indoor environment, ensuring no interference from wind or air currents. The purpose of the insulation was to shield the internal target from minor room fluctuations, allowing us to isolate the camera’s performance. Following the manufacturer’s recommended 15 min warm-up period, the camera was fixed in place, and the temperature of a specific point on the interior wall was recorded every 5 min for 1 hour. This process was repeated six times, with a one-hour cooling-off period between sessions to allow the camera hardware to reset. Our primary goal was to determine if the camera’s internal sensor produced a downward measurement trend over time, rather than to validate absolute temperature accuracy.

### 2.3 Statistical analysis

Graphs and preliminary data visualizations were created using Microsoft Excel and R software (R, version 4.4.2, using RStudio, version 2024.04.2) (26). For the shelter study, ET was analyzed with linear mixed-effects models (PROC MIXED, SAS version 9.4; SAS Institute Inc., Cary, NC). Fixed effects tested in the model included the time (1–3), RT, age, sex, size. Day was included as a random effect. Repeated measurements within dogs were modeled with an autoregressive covariance structure (AR[1]). An autoregressive covariance structure is appropriate when repeated observations closer in time are expected to be more strongly correlated (27). Forward manual selection was used to build the model, variables were retained when they improved model fit (AIC) and were statistically supported at *α* = 0.05. For experiment 2, linear and quadratic linear mixed-effects models were fitted to assess change in IRT spot temperature over the 1-h series. Time since the start of the series was recorded every 5 min and was modeled as the predictor. Both models included a random effect for repetition (group). A significance level of *α* = 0.05 was maintained across all statistical analyses.

## 3 Results

### 3.1 Field study: ocular thermography in shelter dogs

**Figure 2:**
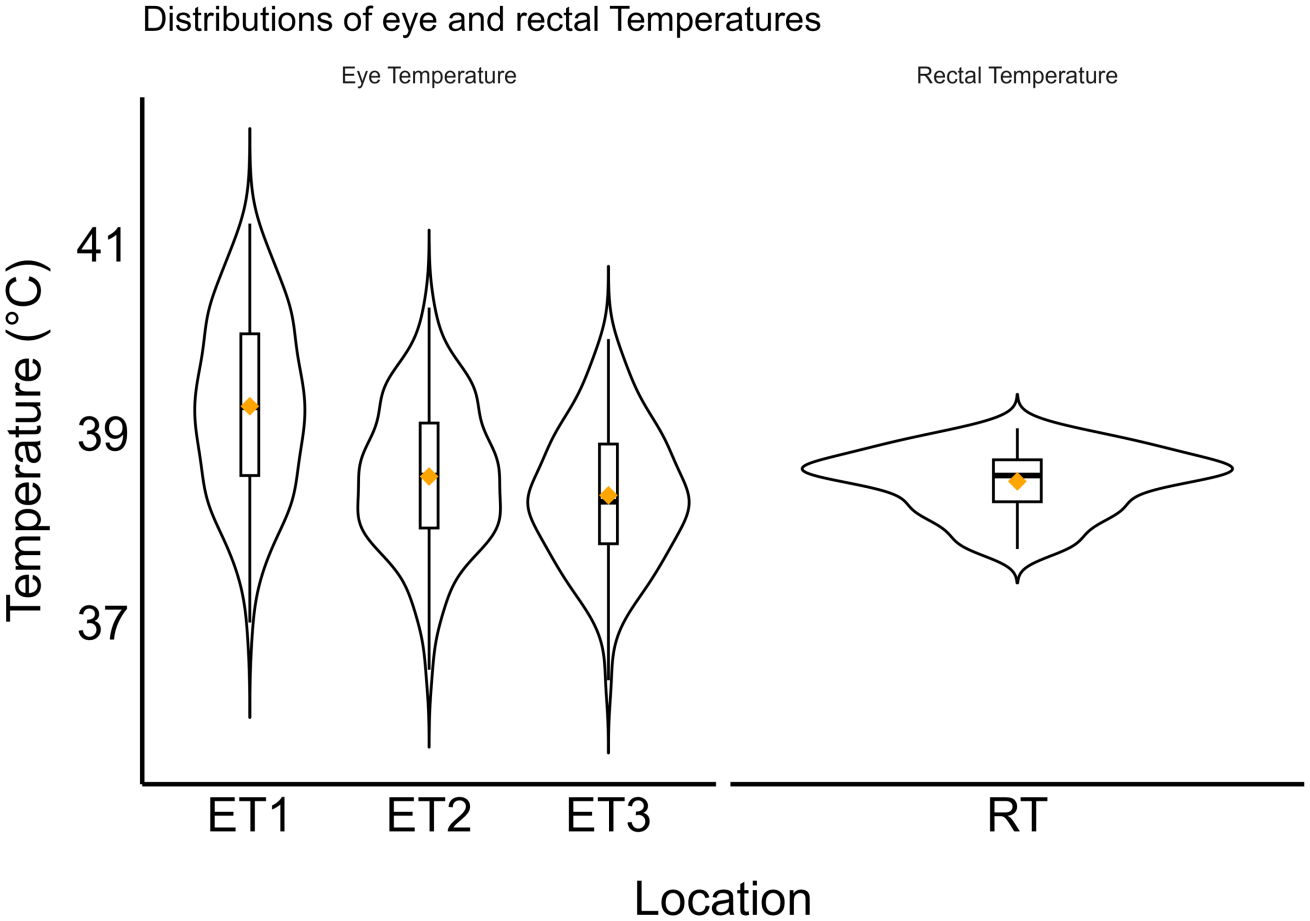
Distributions of maximum eye temperature (ET) at the 3 serial measurements (time 1–3) and rectal temperature (RT). Violin–box plots display the median (horizontal line), interquartile range (box), and range (whiskers); violin width reflects the kernel density.

For this part of our study, a total of 97 observations were recorded, from 57 unique dogs, consisting of 34 females and 23 males, with 23 adults and 34 puppies. Size distribution among unique dogs included 31 small, 21 medium, and 5 large dogs. Since our data collection took place over several different days, the natural turnover of the shelter population meant that some animals were adopted, transferred, or no longer available during subsequent visits. Dogs that remained at the facility over a longer period were evaluated on more than 1 day. This repeated evaluation of the same animals explains why the total number of recorded observations was greater than the number of unique dogs. Throughout the study, the recorded RT of the evaluated dogs ranged from a minimum of 37.8 °C to a maximum of 39.1 °C. The recorded RT measurements showed less dispersion than ET measurements, with values more clustered around the mean, as illustrated in Figure 2.

IRT proved to be a well-received method for temperature measurement, as all dogs accepted the imaging process without signs of stress or discomfort. Relative humidity ranged from 62.5 to 67.0% across the 4 study days (62.5%, 66.4%, 66.4%, and 67% on days 1 through 4, respectively).

**Figure 3:**
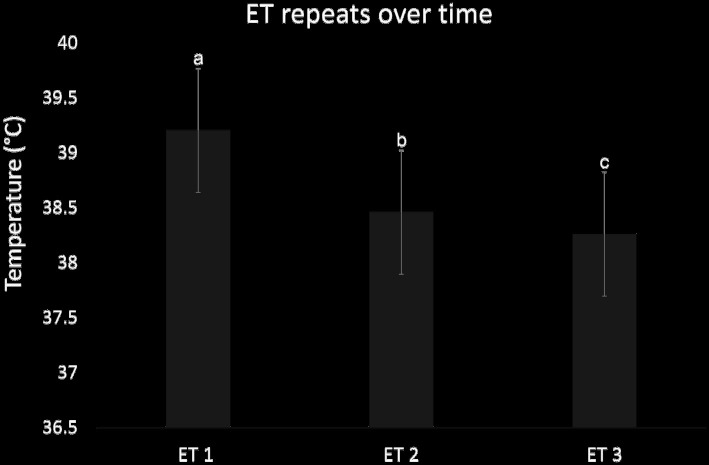
Estimated eye temperature (ET) across repeated measurement groups with 95% confidence intervals. This figure shows estimated ET across repeated measurement groups derived from a linear mixed-effects model, with 95% confidence intervals. Time points that do not share a letter differ in pairwise comparisons (*p* < 0.05).

In the final mixed model, ET was associated with RT, age, and time (Table 1). Notably, within each repeated measurement session, ET decreased across the 3 serial measurements (Figure 3).

**Table 1:** Fixed effects estimates from the final linear mixed model for eye temperature (ET) in shelter dogs (97 dog-day observations from 57 dogs).

Variable	Estimate	95% CI	*p*-value
Intercept	14.6	−5.0 to 34.1	0.099
Time (measurement 1 vs. 3)	0.9	0.8 to 1.1	< 0.001
Time (measurement 2 vs. 3)	0.2	0.1 to 0.3	< 0.001
Time (measurement 3)	Reference	—	—
Rectal temperature (RT), °C	0.6	0.3 to 0.9	< 0.001
Age category (adult vs. puppy)	−0.4	−0.6 to −0.1	< 0.001
Age category (puppy)	Reference	—	—

CI, confidence interval.

Statistical analysis revealed that RT was positively associated with ET (*β* = 0.6, 95% CI, 0.3 to 0.9, *p* < 0.001). Additionally, adult dogs had lower ET than puppies (β = −0.4, 95% CI, −0. 6 to −0.1, *p* = 0.001).

### 3.2 Assessment of device stability

**Figure 4:**
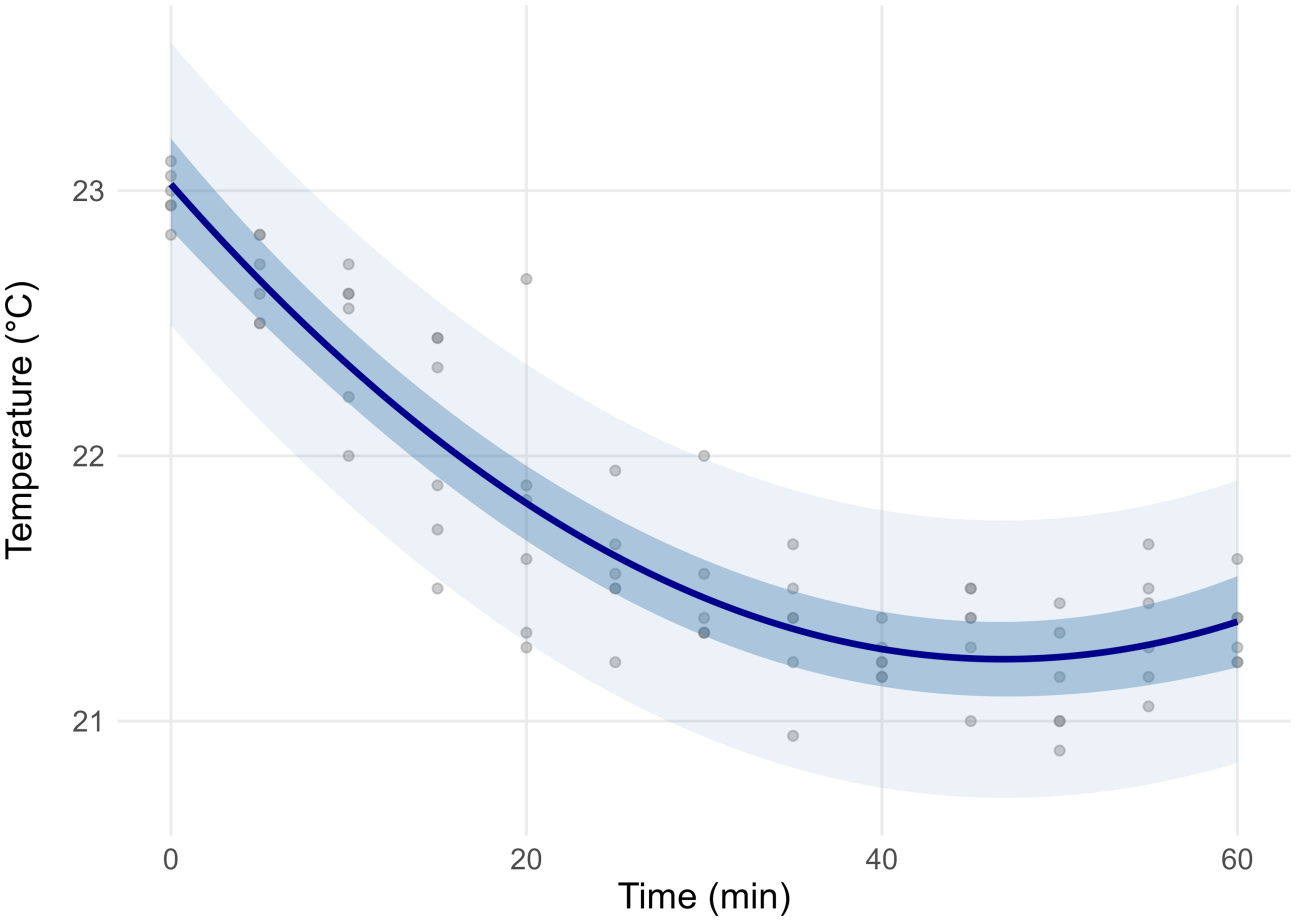
Quadratic regression analysis of temperature over time. The solid dark blue line represents the predicted mean temperature based on a linear mixed-effects model (*y* = 23.02–0.08*x* + 0.0008*x*^2^). The darker shaded area indicates the 95% confidence interval for the fitted mean, while the lighter shaded area represents the 95% prediction interval, accounting for both fixed and random effect (group) variance. Individual data points (*n* = 78) are represented by gray circles.

This experiment evaluated the thermal camera’s temperature measurements in an isolated environment, decreased over time. A model including a quadratic term for time described the pattern of decline, indicating that the temperature decrease was most pronounced during the initial 25 min. After approximately 25 min, the rate of decline diminished over time. Gradually, the rate of temperature decline slowed, eventually reaching a point where no significant further decrease in recorded temperatures was detected (Figure 4; Table 2).

**Table 2:** Fixed effects estimates for time and time^2^ in the insulated box experiment, based on a quadratic mixed model of infrared thermography (IRT) spot temperature.

Variable	Estimate	95% CI	*p* value
Intercept	23.02	22.8 to 23.2	<0.001
Time (min)	−0.08	−0.09 to −0.07	<0.001
Time^2^ (min^2^)	0.0008	0.0007 to 0.001	<0.001

Time measurements were obtained every 5 min. Time^2^ is the squared time term (min^2^).

CI, confidence interval.

## 4 Discussion

This study evaluated whether ET could be obtained under routine shelter conditions and whether ET was associated with RT and dog characteristics. In the shelter dataset, ET was positively associated with RT, and ET differed by age group. The positive relationship between ET and RT makes biological sense when considering how the mammalian body manages heat. Core body temperature is continuously monitored by the hypothalamus, which acts as the brain’s central thermoregulatory part. When internal body temperatures begin to rise, the hypothalamus activates the autonomic nervous system to dissipate the excess heat. It achieves this by forcing the blood vessels near the skin and body surface to widen, a process called vasodilation. Because the ocular area, is heavily supplied by superficial branches of the ophthalmic artery, the increased local blood flow causes the eye surface temperature to rise accordingly (28–30). Therefore, the eye acts as a responsive thermal window that safely reflects internal heat adjustments. Furthermore, adult dogs in our study generally exhibited lower ET compared to puppies. This variation might get influenced by two main physiological differences. First, puppies possess a larger surface area-to-mass ratio and typically maintain higher baseline metabolic rates, which keeps their surface heat warmer. Second, young puppies possess a physically immature hypothalamic heat-regulation network, meaning their autonomic control of surface blood vessels is not as highly refined as in mature adult dogs, leading to differing surface temperature presentations (31–33). In the shelter dataset, ET was positively associated with RT, and ET differed by age group. Similarly, Elias et al. reported that older dogs exhibited a smaller increase in ET following races compared to younger ones (34). Although, Travain et al. (21) noted that during some part of their experiments, dogs exhibited some level of discomfort with the thermographic tool, in our experiment the thermal camera did not appear to cause any noticeable distress in the dogs. We considered the dogs to be free of stress because, during thermal imaging, they did not show fearful behaviors such as attempting to escape, trembling, barking, or acting aggressive.

In field study, we found that IRT readings decreased consistently with each time period. We conducted device stability’s assessment study to understand this phenomenon. It is possible that a dog’s initial excitement when unfamiliar individuals approached their kennel caused a slight temporary increase in ET during the very first reading (21). However, because our controlled environment assessment also showed a similar dropping pattern over time, we believe the internal camera measurement error is the main reason for the decreasing values we observed across the 10-min intervals. The results of this device validation study demonstrated that the specific camera we used for data collection in this study, did not maintain consistent accuracy over time. This finding suggests that the camera itself introduced a significant source of variation in the measurements, potentially affecting the overall precision and validity of the recorded data. This pattern suggests that the decline may result from various reasons such as camera heat dissipation system. While thermal device instability is a recognized technical phenomenon (24), to our knowledge, this study is the first to systematically quantify how such device-related measurement drift can manifest during veterinary settings. Our findings highlight that this equipment-related variability can be easily misidentified as physiological change, emphasizing the necessity of stability checks in veterinary IRT research. Given these findings, we emphasize that future thermographic studies should prioritize the use of cameras with validated temporal stability to ensure that observed temperature fluctuations reflect biological responses rather than equipment drift.

In field study, we accounted for the effect of time in the multivariable model, allowing us to report the effects of RT and age on ET. Therefore, even though we found that the camera values drifted downward over time, our statistical model protected our main biological findings. Because we included the measurement time (first, second, and third reading) as an independent fixed effect in our linear mixed-effects model, the mathematical analysis automatically adjusted for this device drift. This allowed the model to successfully separate camera error and evaluate how RT and age affected ET.

A limitation of our research was the span of recorded body temperatures, which reflects the generally healthy nature of this specific shelter population. In linear regression models, evaluating a highly restricted data range limits the available variance. Under these conditions, minor physiological fluctuations or device measurement errors become proportionally larger compared to the true biological signal. Because of this restricted range, the true magnitude of the relationship between ET and RT might be partially obscured. Studies evaluating animals with abnormal temperature, have been able to report diagnostic performance when a wider thermal range is present (35). Future studies involving clinically sick dogs with high fevers or hypothermia will provide a wider temperature range, which will be essential to fully define the mathematical association between rectal and surface body temperatures.

We intentionally utilized the maximum recorded temperature from the ocular area rather than targeting a specific anatomical structure. In a realistic veterinary setting, animals move, and staff members might not have the time or training to capture images at mathematically perfect angles or distances. Extracting the maximum temperature is a fast, standardized approach that removes human error and mimics the practical constraints of daily rounds.

In conclusion, ET remained positively associated with RT and differed by age group in this dog population. Our mixed model estimated that every 1 °C increase in RT is linked to an average 0.6 °C increase in ET, while adult dogs exhibited an average ET that was 0.4 °C lower than puppies. Eye thermography has the potential to serve as a non-invasive adjunct tool to standard clinical assessments, but its performance must be further validated in patient populations presenting with a wider range of clinically abnormal temperatures. Furthermore, the thermal camera used in this study showed time-dependent recording drift. Researchers and practitioners must test thermal devices for hardware stability, as device-related drift can easily skew repeated measurements if not mathematically accounted for during analysis.

## Data Availability

The raw data supporting the conclusions of this article will be made available by the authors, without undue reservation.

## References

[1] Lopedote M Valentini S Musella V Vilar JM Spinella G. Changes in pulse rate, respiratory rate and rectal temperature in working dogs before and after three different field trials. Animals. (2020) 10:4. doi: 10.3390/ani10040733, 32340191 PMC7222833

[2] Piccione G Fazio F Giudice E Refinetti R. Body size and the daily rhythm of body temperature in dogs. J Therm Biol. (2009) 34:171–5. doi: 10.1016/j.jtherbio.2009.01.004

[3] Bruchim Y Klement E Saragusty J Finkeilstein E Kass P Aroch I. Heat stroke in dogs: a retrospective study of 54 cases (1999–2004) and analysis of risk factors for death. J Vet Intern Med. (2006) 20:38–46. doi: 10.1111/j.1939-1676.2006.tb02821.x, 16496921

[4] Burban LS (2018) Environmental factors that affect kenneled shelter dogs in Connecticut. Available online at: https://www.proquest.com/docview/3073205865/abstract/8FF6F60C06974117PQ/1 (Accessed February 17, 2025)

[5] Azeez OM Olaifa FH Adah AS Basiru A Akorede GJ Ambali HM . Effect of heat stress on vital and hematobiochemical parameters of healthy dogs. Vet World. (2022) 15:722–7. doi: 10.14202/vetworld.2022.722-727, 35497950 PMC9047135

[6] Lavan R Knesl O. Prevalence of canine infectious respiratory pathogens in asymptomatic dogs presented at US animal shelters. J Small Anim Pract. (2015) 56:572–6. doi: 10.1111/jsap.12389, 26199194 PMC7166506

[7] Dallmann R Steinlechner S von Hörsten S Karl T. Stress-induced hyperthermia in the rat: comparison of classical and novel recording methods. Lab Anim. (2006) 40:186–93. doi: 10.1258/002367706776319015, 16600078

[8] Stewart M Webster JR Schaefer AL Cook NJ Scott SL. Infrared thermography as a non-invasive tool to study animal welfare. Anim Welf. (2005) 14:319–25. doi: 10.1017/S096272860002964X

[9] Im SWK Chow K Chau PY. Rectal thermometer mediated cross-infection with *Salmonella wandsworth* in a paediatric ward. J Hosp Infect. (1981) 2:171–4. doi: 10.1016/0195-6701(81)90026-8, 6174580

[10] Weese JS Caldwell F Willey BM Kreiswirth BN McGeer A Rousseau J . An outbreak of methicillin-resistant *Staphylococcus aureus* skin infections resulting from horse to human transmission in a veterinary hospital. Vet Microbiol. (2006) 114:160–4. doi: 10.1016/j.vetmic.2005.11.054, 16384660

[11] McCafferty DJ. The value of infrared thermography for research on mammals: previous applications and future directions. Mammal Rev. (2007) 37:207–23. doi: 10.1111/j.1365-2907.2007.00111.x

[12] Schaefer AL Cook NJ Church JS Basarab J Perry B Miller C . The use of infrared thermography as an early indicator of bovine respiratory disease complex in calves. Res Vet Sci. (2007) 83:376–84. doi: 10.1016/j.rvsc.2007.01.008, 17349665 PMC7111866

[13] Lahiri BB Bagavathiappan S Jayakumar T Philip J. Medical applications of infrared thermography: a review. Infrared Phys Technol. (2012) 55:221–35. doi: 10.1016/j.infrared.2012.03.007, 32288544 PMC7110787

[14] Mota-Rojas D Pereira A Wang D Marinez-Burnes J Casas A Dominguez A (2022) Clinical applications and factors involved in validating thermal windows used in infrared thermography in cattle and river buffalo to assess health and productivity. Available online at: https://agris.fao.org/search/en/providers/125169/records/67659ecb829d7ef70f492832 (Accessed April 25, 2026) 10.3390/ani11082247 PMC8388381 34438705

[15] Zaha C Cărpinișan L Schuszler L Paula N Căsălean T Florea T . Thermographic scan of the thoracolumbar area in dogs with acute intervertebral disc extrusion (IVDE): a retrospective study. Life. (2025) 15:1. doi: 10.3390/life15010068, 39860008 PMC11766613

[16] Casas-Alvarado A Ogi A Villanueva-García D Martínez-Burnes J Hernández-Avalos I Olmos-Hernández A . Application of infrared thermography in the rehabilitation of patients in veterinary medicine. Animals. (2024) 14:5. doi: 10.3390/ani14050696, 38473082 PMC10930678

[17] Cugmas B Šušterič P Gorenjec NR Plavec T. Comparison between rectal and body surface temperature in dogs by the calibrated infrared thermometer. Vet Animal Sci. (2020) 9:100120. doi: 10.1016/j.vas.2020.100120, 32734121 PMC7386665

[18] Zanghi BM. Eye and ear temperature using infrared thermography are related to rectal temperature in dogs at rest or with exercise. Front Vet Sci. (2016) 3:3. doi: 10.3389/fvets.2016.00111, 28066775 PMC5165259

[19] Yanmaz LE Doğan E Okumuş Z Şenocak MG Yildirim F. Kangal Irkı Köpeklerde Kulak, Göz ve Rektal Isıların Karşılaştırılması. Kafkas Univ Vet Fak Derg. (2015). doi: 10.9775/kvfd.2015.13037

[20] Okur S Değirmençay Ş Senocak MG Ersöz U Yanmaz LE Gölgeli A. The agreement of rectal temperature with gingival, ocular and metacarpal pad temperatures in clinically healthy dogs. N Z Vet J. (2022) 70:159–64. doi: 10.1080/00480169.2021.2017373, 34890521

[21] Travain T Colombo ES Heinzl E Bellucci D Prato Previde E Valsecchi P. Hot dogs: thermography in the assessment of stress in dogs (*Canis familiaris*)—a pilot study. J Vet Behav. (2015) 10:17–23. doi: 10.1016/j.jveb.2014.11.003

[22] Casas-Alvarado A Martínez-Burnes J Mora-Medina P Hernández-Avalos I Domínguez-Oliva A Lezama-García K . Thermal and circulatory changes in diverse body regions in dogs and cats evaluated by infrared thermography. Animals. (2022) 12:6. doi: 10.3390/ani12060789, 35327185 PMC8944468

[23] Kwon CJ Brundage CM. Quantifying body surface temperature differences in canine coat types using infrared thermography. J Therm Biol. (2019) 82:18–22. doi: 10.1016/j.jtherbio.2019.03.004, 31128646

[24] Minkina W Dudzik S. Infrared Thermography: Errors and Uncertainties. Hoboken, NJ: John Wiley & Sons (2009).

[25] Church JS Hegadoren PR Paetkau MJ Miller CC Regev-Shoshani G Schaefer AL . Influence of environmental factors on infrared eye temperature measurements in cattle. Res Vet Sci. (2014) 96:220–6. doi: 10.1016/j.rvsc.2013.11.006, 24290729

[26] R Project (2025) R: The R Project for Statistical Computing. Available online at: https://www.r-project.org/ (Accessed August 5, 2025)

[27] Littell RC Milliken GA Stroup WW Wolfinger RD Oliver S. SAS for Mixed Models. Cary, NC: SAS Publishing (2006).

[28] Flammer J Konieczka K Flammer AJ. The primary vascular dysregulation syndrome: implications for eye diseases. EPMA J. (2013) 4:14. doi: 10.1186/1878-5085-4-14, 23742177 PMC3693953

[29] Mota-Rojas D Ghezzi MD Hernández-Ávalos I Domínguez-Oliva A Casas-Alvarado A Lendez PA . Hypothalamic neuromodulation of hypothermia in domestic animals. Animals. (2024) 14:513. doi: 10.3390/ani14030513, 38338158 PMC10854546

[30] Trachtman JN. Vision and the hypothalamus. Optometry. (2010) 81:100–15. doi: 10.1016/j.optm.2009.07.016, 20152784

[31] Grundy SA. Clinically relevant physiology of the neonate. Vet Clin North Am Small Anim Pract. (2006) 36:443–59. doi: 10.1016/j.cvsm.2005.12.002, 16564408

[32] Moon PF Massat BJ Pascoe PJ. Neonatal critical care. Vet Clin North Am Small Anim Pract. (2001) 31:343–67. doi: 10.1016/S0195-5616(01)50209-0, 11265496

[33] Münnich A Küchenmeister U. Causes, diagnosis and therapy of common diseases in neonatal puppies in the first days of life: cornerstones of practical approach. Reprod Domest Anim. (2014) 49:64–74. doi: 10.1111/rda.12329, 24947863

[34] Elias B Starling M Wilson B McGreevy P. Influences on infrared thermography of the canine eye in relation to the stress and arousal of racing greyhounds. Animals. (2021) 11:103. doi: 10.3390/ani11010103, 33419209 PMC7825601

[35] Schaefer AL Cook NJ Bench C Chabot JB Colyn J Liu T . The non-invasive and automated detection of bovine respiratory disease onset in receiver calves using infrared thermography. Res Vet Sci. (2012) 93:928–35. doi: 10.1016/j.rvsc.2011.09.021, 22055252 PMC7111736

